# Shared genetic variants between serum levels of high-density lipoprotein cholesterol and wheezing in a cohort of children from Cyprus

**DOI:** 10.1186/s13052-016-0276-1

**Published:** 2016-07-13

**Authors:** Panayiotis K. Yiallouros, Panayiotis Kouis, Ourania Kolokotroni, Sonia Youhanna, Savvas C. Savva, Kleanthi Dima, Aikaterini Zerva, Danielle Platt, Nicos Middleton, Pierre Zalloua

**Affiliations:** Medical School, University of Cyprus, Nicosia, Cyprus; Cyprus International Institute for Environmental & Public Health in Association with Harvard School of Public Health, Cyprus University of Technology, Limassol, Cyprus; Department of Nursing, School of Health Sciences, Cyprus University of Technology, Limassol, Cyprus; St George University of London Medical School at the University of Nicosia, Nicosia, Cyprus; Lebanese American University, School of Medicine, Beirut, Lebanon; Research and Education Institute of Child Health, Nicosia, Cyprus; Department of Biochemistry, Attikon University Hospital, Athens, Greece

**Keywords:** Asthma, Children, High density lipoprotein cholesterol, Polymorphisms, Genotypes

## Abstract

**Background:**

In a cohort of children in Cyprus, we recently reported low levels of high density lipoprotein cholesterol (HDL-C) to be associated with asthma. We examined whether genetic polymorphisms that were previously linked individually to asthma, obesity, or HDL-C are associated with both asthma and HDL-C levels in the Cyprus cohort.

**Methods:**

We assessed genotypes frequencies in current-wheezers (*n* = 190) and non-asthmatic controls (*n* = 671) and HDL-C levels across several genotypes. Binary logistic regression models were used to assess the effect of genotypes on wheezing risk and examined whether this effect is carried out through changes of HDL–C.

**Results:**

Of the 16 polymorphisms tested, two polymorphisms *TNFa rs3093664* and *PRKCA rs9892651* presented significant differences in genotype distribution among current-wheezers and controls. Higher HDL-C levels were noted in carriers of genotype GG of polymorphism *TNFa rs3093664* that was protective for wheezing Vs AG and AA genotypes (65.3 Vs 51.8 and 53.3 mg/dl, *p*-value < 0.001 and *p*-value for trend = 0.028). In polymorphism *PRKCA rs9892651*, HDL-C levels were lower in carriers of CC and TC genotypes that were more frequent in current-wheezers Vs TT genotype (52.2 and 52.7 Vs 55.2 mg/dl, *p*-value = 0.042 and *p*-value for trend = 0.02). The association of *TNFa rs3093664* with wheezing is partly mediated by its effect on HDL-C whereas association of *PRKCA rs9892651* with wheezing appeared to be independent of HDL-C.

**Conclusions:**

We found evidence that two SNPs located in different genetic loci, are associated with both wheezing and HDL-C levels, although more studies in other populations are needed to confirm our results.

**Electronic supplementary material:**

The online version of this article (doi:10.1186/s13052-016-0276-1) contains supplementary material, which is available to authorized users.

## Background

High density lipoprotein cholesterol (HDL-C) is widely known to have systemic and vascular anti-inflammatory properties [1] and, as shown in vitro, also anti-inflammatory effects consistent with a protective role in asthma [2]. In a cohort of 3982 children in Cyprus, we recently reported low levels of HDL-C at age 11–12 years to be associated with active asthma five years later in adolescence, suggesting a potential role of this lipoprotein in the pathogenesis of pediatric asthma [3]. Obesity is also widely accepted to be related to asthma [4] as well as with low serum HDL-C levels [5] and consequently it may play a confounding or mediating role in the asthma – HDL-C association.

Numerous individual studies confirmed the implication of genetic factors in the development of asthma [6], obesity [7] or low HDL-C [8]. In addition, epidemiological studies in twins have suggested that the association of asthma and allergy with obesity or low HDL-C levels could, at least in part, be explained by the presence of shared genetic factors [9]. In the case of asthma and obesity, overlapping genomic regions for common susceptibility genes on chromosomes 5, 6, 11 and 12 have been identified by linkage analysis studies [10]. Specifically, some of the most known candidate genes and genetic loci for asthma and allergy [6, 11] have already been linked [12] to the pathogenesis of obesity such as the tumor necrosis factor alpha (*TNF-a)* gene [12], the protein kinase C alpha (*PRKCA)* gene [13] and β2‐adrenergic receptor (*ADRB2*) [14]. To date however, no common genetic loci were reported to be associated with asthma and HDL-C levels.

In this study, we selected 16 single nucleotide polymorphisms (SNPs) located in genes that were linked to asthma and/or obesity or HDL-C and examined in the Cyprus cohort of children a) whether they were associated with both asthma and HDL-C levels and b) explored, in the SNPs that were associated with both conditions, whether the association with asthma is mediated through changes in the levels of HDL-C.

## Methods

### Study population

**Fig. 1:**
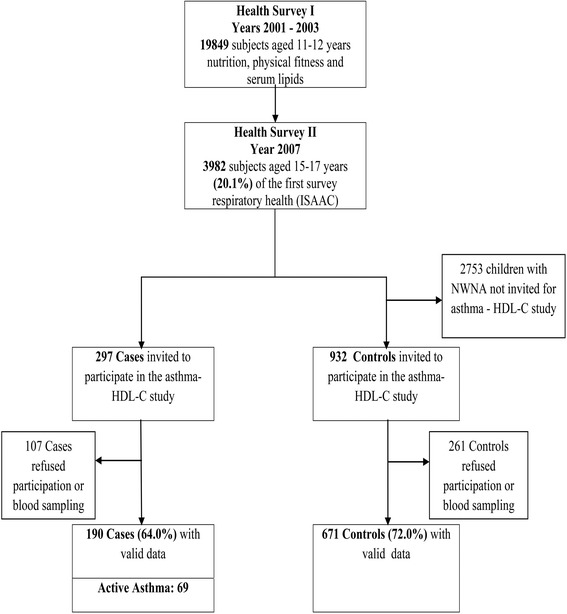
Course of the study: From the 19,849 participants (11–12 years) in Survey 1, a random sample of 3982 adolescents (15–17 years) participated in Survey 2. All (*n* = 297) subjects from the 3982 sample, who reported wheezing in the last 12 months were invited to participate as cases. A sample of 932 children with no diagnosis of asthma and no wheezing in the past 12 months were invited to participate as controls

The participants of this study were selected from a cohort of 3982 children who participated in two large school-based health surveys in Cyprus (Fig. 1). The first survey spanned years 2001–2003 and involved all children (*n* = 19,849) attending the 6th grade from all primary schools across Cyprus and focused on nutrition and physical fitness. The second survey was conducted in year 2007, followed-up 3982 of the subjects who took part in the first survey (20.1 %) and focused on respiratory health (International Study of Asthma and Allergies in Childhood – ISAAC) and risk factors for asthma. In a case-control design, we invited all those from the 3982 children who on the second survey (ISAAC questionnaire) reported wheezing in the past 12 months (*n* = 297) to participate in this study along with a triplicate number of healthy controls (*n* = 932).

### Phenotype description

Current wheezers (CUWH) were named those who reported wheezing in the past 12 months. For the purpose of performing a sensitivity analysis, the case definition was further refined to Active Asthma (ACAS), if also there was report of diagnosis of asthma ever. Controls were selected amongst the 3982 adolescents who did not report any wheezing or asthma ever (Never Wheezers Never Asthmatics - NWNA). NWNA were selected using a stratified random sampling approach in order to increase the probability of selection of children at the extremes of Body Mass Index (BMI) change between childhood and adolescence, in line with the scope of another study on the relation of adiposity and serum lipids with asthma [15]. The full scale of BMI change (and BMI) was represented in the final sample, just in slightly different proportions compared to the general population i.e. there was slight over-representation of participants whose BMI increased or decreased between childhood and adolescence and, equivalently slight under-representation of children whose BMI remained stable over this period. In all analyses normalized sampling weights based on inverse probability of selection from the original population were used.

In the asthmatic groups, information was also obtained regarding the presence of exercise induced wheezing and admissions ever in hospital for an asthma attack. IgE sensitization was assessed with skin prick tests (SPT) to 8 common aeroallergens (Greer, USA), performed and interpreted according to the GA2LEN recommendations [16]. Children with a positive SPT to any of the 8 allergens tested were considered sensitized to IgE. Asthmatic children had measurements of forced expiratory volume in one second (FEV1) (Vitallograph, UK) and values were expressed as percent of predicted [17].

The adolescents had anthropometric measurements and assessments of serum total cholesterol (TC), low density lipoprotein cholesterol (LDL-C), HDL-C and triglycerides (TG) after a 12-h fast. We calculated BMI (kg/m2) and expressed it as age- and gender-specific z-scores based on the United States’ Centers for Disease Control 2000 growth charts [18]. Lipids were determined with the ILab 600 analyzer using ILab Chemistry systems (Instrumentation Laboratory SpA, Monza, Milano, Italy). The assays’ coefficients of variation were 2.08 % for TC, 1.53 % for HDL-C, 1.15 % for LDL-C and 2.45 % for TG.

### SNPs selection, DNA extraction and genotyping

Using the NCBI PubMed Entrez database, a total of 16 SNPs in ten genes that were previously reported [12–14, 19–30] to be associated individually with asthma, obesity or HDL-C were selected for genotyping (Table 1).

**Table 1:** The 16 investigated SNPs with reported links with asthma, obesity, or HDL-C

Gene	Chromosome	SNPs genotyped	dbSNPrs#	Alleles	Published literature reference
IL1R1	2	1	rs1420101	C > T	Gudbjartsson 2009 [19]
ACP1	2	1	rs12714402	G > A	Bottini 2007 [20]
GNPDA2	4	1	rs10938397	A > G	Melen 2010 [21]
IL13	5	1	rs20541	C > T	Cui 2012 [22]
ADRB2	5	3	rs1800888	C > T	Hall 2006 [23]
			rs1042714	C > G	Park 2008 [24]
			rs1042713	G > A	Garenc 2002 [25]
TNF-a	6	3	rs3093664	A > G	Joubert 2011 [26]
			rs1800629	G > A	Thomas 2001 [27]
			rs361525	G > A	Castro 2009; Joffe 2012 [12, 28]
LEP	7	1	rs2167270	G > A	Ichihara 2008 [29]
ACE	17	2	rs4343	A > G	Rankinen 2006 [14]
			rs4311	C > T	
GSDMB	17	1	rs7216389	T > C	Halapi 2010 [30]
PRKCA	17	2	rs9892651	T > C	Murphy 2009 [13]
			rs9901804	G > A	

Venous blood specimens were collected from participants in EDTA treated tubes that were transported on ice to the laboratory. DNA was extracted from peripheral blood lymphocytes using the standard phenol extraction method, and stored at −70 °C until genotyping. Genotyping was carried out using TaqMan Real-time PCR assay. Thirty ng of genomic DNA was used for each reaction and the end-point PCR was carried out on the ABI primer 7900 HT (Applied Biosystems, Foster City, CA).

### Statistical analysis

Differences in participant characteristics between study-groups were investigated in a pairwise manner (NWNA versus CUWH and NWNA versus ACAS) using chi square test in the case of categorical variables and *t*-test in the case of continuous variables. Furthermore, chi-squared testing was used for Hardy-Weinberg (HW) equilibrium determination and for the investigation of genotypes and allele frequencies associations with study groups. Mean HDL-C levels were compared across genotypes using the one-way ANOVA test and *p* values and *p* values for trend were reported. For SNPs that presented significant effects, two binary logistic regression models were used a) to assess the effect of genotypes on asthma risk and b) examine whether this effect is carried out through the change of HDL–C, after adjusting for age and sex (Model 1), age, sex and BMI (Model 2) and age, sex, BMI and HDL-C levels (Model 3). The genotype with the most protective effect for asthma was chosen as the reference in all cases. Binary logistic regression analysis was performed only among NWNA and CUWH as the ACAS group was very small and for most SNPs, at least one cell had no cases. Analysis was performed with SPSS statistical package, version 20.0 (IBM, SPSS Inc., Chicago, IL).

## Results

A total of 190 subjects with CUWH (of whom 69 ACAS) and 671 NWNA participated in the study. This corresponds to 64 and 72 % response rates respectively of the targeted population for recruitment (Fig. 1). Participants were aged 17 years (SD 0.6) and the three groups did not differ significantly in terms of age, gender and BMI (Table 2). Mean HDL-C levels in the ACAS and CUWH were significantly lower than in the controls (47.9 and 49.1 Vs 53.4 mg/dl; *p* < 0.001 for both) (Table 2). Mean serum levels of TC, LDL-C and TG were similar in the three groups (results not shown in detail). The frequency of atopic sensitization was 52.0 % in ACAS and 41.4 % in CUWH (*p*-value = 0.073), whereas FEV1 was normal and not different between them (97.6 % Vs 99.1 % of predicted; *p*-value = 0.332) indicating that they were clinically stable. ACAS were experiencing more frequently exercise induced wheezing (83.6 % vs 67.6 %, *p*-value < 0.001) and lifetime hospitalizations for asthma attack (18.8 % vs 9.0 %, *p*-value = 0.003) than the CUWH (Table 2). Results of minor allele frequencies and HW equilibrium for genotype distribution are presented in Additional file 1: Table S1.

**Table 2:** Characteristics of study population according to asthma status

	NWNA (Controls)	CUWH (Current Wheezers)	*p-*value^c^	ACAS (Active Asthmatics)	*p-*value^d^
	(*n* = 671)	(*n* = 190)		(*n* = 69)	
Age (yrs)^a^	16.95 (15.91–18.00)	17.00 (16.00–18.11)	0.309	16.91 (15.91–18.07)	0.567
Sex (% female)^b^	57.4 %	56.9 %	0.914	56.5 %	0.894
BMI z scores^a^	0.358 ( −2.13–2.16)	0.288 ( −1.73–2.20)	0.396	0.340 ( −1.87–2.37)	0.896
HDL (mg/dl)^a^	53.4 (30.00–86.50)	49.1 (30.00–71.55)	<0.001	47.9 (31.73–73.75)	<0.001
Atopic Sensitization (% positive)^b^	-	41.4 %	-	52.0 %	0.073^e^
FEV1 (% predicted)^a^	-	99.1 (72.9–130.5)	-	97.6 (62.0–125.6)	0.332^e^
Exercise Induced Wheezing (% positive)^b^	-	67.6 %	-	83.6 %	<0.001^e^
Hospitalization (% positive)^b^	-	9.0 %	-	18.8 %	0.003^e^

^a^Mean and 95 % CI, Independent sample *t* test for equality of means (2-sited significance)

^b^Percentage, *χ* ^2^ test (asymptomatic 2-sited significance)

^c^Comparison between NWNA and CUWH

^d^Comparison between NWNA and ACAS

^e^Comparison between CUWH and ACAS

### SNPs genotype distribution in CUWH Vs NWNA and HDL-C levels

Distribution of the genotypes of the 16 polymorphisms in CUWH Vs NWNA and the corresponding HDL-C levels can be found in Additional file 2: Table S2. Two polymorphisms, *TNFa rs3093664* and *PRKCA rs9892651* presented statistically significant and *ADRB2 rs1800888* nearly significant differences in genotype distribution among NWNA and CUWH and are presented separately at Table 3.

**Table 3:** SNPs genotype distribution in Current Wheezers (CUWH) Vs Controls (NWNA) and HDL-C levels

SNP (genotype)	NWNA (n, %)	CUWH (n, %)	*χ* ^2^	*p* value	*p* trend	HDL mg/dl (mean, 95 % CI)	F	*p* value	*p* trend
*ADRB2 rs1800888*									
CC	609 (95 %)	175 (97.8 %)				53.3 (52.3–54.2)			
CT	32 (5.0 %)	4 (2.2 %)	2.535	0.076^b^	-	57.2 (51.7–62.8)	2.840	0.092^c^	-
*TNFa rs3093664*									
AA	500 (79.6)	134 (76.1)				53.3 (52.2–54.3)			
AG	102 (16.2)	40 (22.7)				51.7 (49.4–54.1)			
GG	26 (4.1)	2 (1.1)	7.047	0.03^a^	0.912	65.3 (59.2–71.4)	11.787	>0.001^c^	0.028
*PRKCA rs9892651*									
TT	230 (37.0)	52 (30.2)				55.2 (53.4–56.9)			
CT	302 (48,6)	87 (50.6)				52.7 (51.4–54.1)			
CC	89 (14.3)	33 (19.2)	3.921	0.141^a^	0.048	52.2 (50.0–54.5)	3.179	0.042^c^	0.020

^a^ *χ* ^2^ test (asymptomatic 2- sided significance)

^b^Fischer Exact test (exact 1-sided significance)

^c^Comparisons of means (One way ANOVA)

The larger differences were exhibited in the genotype distribution of *TNFa rs3093664* polymorphism, where the rare GG genotype appeared to offer a protective effect with a frequency of 4.1 % Vs 1.1 % in NWNA and CUWH respectively (*p*-value = 0.03, *p*-value for trend = 0.912). Significantly higher HDL-C levels were noted in subjects with GG genotype as opposed to those in AG and AA genotype carriers (65.3 mg/dl Vs 51.8 mg/dl and 53.3 mg/dl, *p*-value < 0.001 and *p*-value for trend = 0.028) (Table 3). The genotypes of polymorphism *PRKCA rs9892651* were also found to be differentially distributed with the frequency of genotype CC to be higher in CUWH (19.2 %) compared to NWNA (14.3 %) (*p*-value = 0.141, *p*-value for trend = 0.048). HDL-C levels were also significantly lower in subjects with the CC and TC genotype compared to TT genotype (52.2 mg/dl and 52.7 mg/dl Vs 55.2 mg/dl, *p*-value = 0.042 and *p*-value for trend = 0.02).

The only other SNP that displayed nearly significant differences in genotype frequencies was *ADRB2 rs1800888*, where the heterozygous genotype CT had a higher frequency in NWNA compared to CUWH (5 % Vs 2.2 %, *p*-value = 0.076) thus exhibiting a protective effect compared with the homozygote CC genotype. HDL-C levels in the protective CT genotype carriers were not significantly higher at the 5 % level to those in CC, probably due to the small size of the CT genotype group (57.24 mg/dl Vs 53.28 mg/dl, *p*-value = 0.092).

### SNPs genotype distribution in ACAS Vs NWNA and HDL-C levels

As a sensitivity analysis, the same investigations were performed between the more strictly defined asthmatic phenotype ACAS and NWNA. In general, the magnitude and direction of the relationships of the three SNPs genotypes in ACAS and NWNA remained consistent with the findings of the NWNA Vs CUWH analysis although these were not always significant probably due to the smaller size of the ACAS study-group. A detailed description of these findings is presented with Additional file 3: Table S3. Allelic associations of the 16 tested SNPs with study groups were not statistically different and are presented in Additional file 4: Table S4.

### Associations of SNPs genotypes with CUWH and the mediating role of HDL-C levels

Associations of *rs3093664, rs9892651* and *rs1800888* genotypes with CUWH were explored in binary logistic regression models adjusted for age and sex (Model 1) age, sex and BMI (Model 2) and with the inclusion of HDL-C levels (Model 3) (Table 4). The estimates in all comparisons between Models 1 and 2 were very similar, thus excluding any confounding or mediating effect of BMI. In particular, for the *TNF-a rs3093664* polymorphism, the Odds Ratios (ORs) of having CUWH for genotypes AA and AG against GG were 3.35 (95 % CI 0.78, 14.35) and 4.92 (95 % CI 1.11, 21.8) respectively (Model 2). After further adjusting for HDL-C (Model 3), the calculated ORs of CUWH were attenuated by approximately 40 % (OR = 2.44, 95 % CI 0.56, 10.58 for genotype AA and OR = 3.43 95 % CI 0.76, 15.53 for genotype AG), thus indicating that the protective effect of GG for CUWH is mediated to an important extent through HDL-C levels.

**Table 4:** Associations of *TNFa, PRKCA* and *ADRB2* genotypes with Current Wheezers and the mediating role of HDL-C levels

SNP (genotype)	Model 1 OR^a^ (95 % CI)	*p*-value	Model 2 OR^b^ (95 % CI)	*p*- value	Model 3 OR^c^ (95 % CI)	*p*- value
*ADRB2 rs1800888*						
CT	1.00		1.00		1.00	
CC	2.18 (0.76–6.29)	0.148	2.15 (0.75–6.21)	0.156	1.98 (0.68–5.79)	0.212
*TNFa rs3093664*						
GG	1.00		1.00		1.00	
AG	5.02 (1.13–22.25)	0.034	4.92 (1.11–21.80)	0.036	3.43 (0.76–15.53)	0.110
AA	3.39 (0.79–14.49)	0.100	3.35 (0.78–14.33)	0.104	2.44 (0.56–10.58)	0.239
*PRKCA rs9892651*						
TT	1.00		1.00		1.00	
CT	1.25 (0.85–1.84)	0.255	1.26 (0.85–1.85)	0.250	1.17 (0.79–1.73)	0.448
CC	1.623 (0.98–2.68)	0.058	1.63 (0.99–2.69)	0.056	1.52 (0.91–2.53)	0.110

^a^Model 1 adjusted for age & sex

^b^Model 2 adjusted for age, sex & BMI z scores

^c^Model 3 adjusted for age, sex, BMI z scores & HDL-C levels

In the case of *PRKCA rs9892651,* the ORs for genotype CC carriers to have CUWH compared to TT (OR = 1.63, 95 % CI 0.99, 2.69) and CT (OR = 1.26, 95 % CI 0.85, 1.85) were slightly short of significance in Model 2. In Model 3, these estimates were only moderately attenuated for TT (OR = 1.52, 95 % CI 0.91, 2.53) and CT (OR = 1.17, 95 % CI 0.79, 1.73). Thus *PRKCA rs9892651* association with wheezing appeared to be independent of HDL-C levels.

## Discussion

In this case-control study, we examined the effect of 16 genetic variants on asthma phenotypes and identified two SNPs that were simultaneously associated with the wheezing and HDL-C levels. Although many studies have examined separately the relationship of genetic loci with respiratory outcomes, only few have searched for common genetic variants between asthma and metabolic markers [12, 13, 21, 31]. Skaaby et al., studied loss-of-function mutations in the filaggrin gene, which are known to be associated with asthma and allergies and higher vitamin D, and found higher vitamin D status to be associated with higher HDL-C levels [32], suggesting an indirect potential genetic link between asthma and allergies with high HDL-C levels. In our cohort, we have not examined polymorphisms in the filaggrin gene, but to the best of our knowledge, this is the only study so far which, in the same population, assessed simultaneously the direct association of selected SNPs with wheezing and HDL-C levels. Interestingly, in our study the direction of the association of wheezing and HDL-C levels is in the opposite direction from the one implied in the Skaaby et al. study. More importantly, our results provide preliminary evidence that the association of *TNFa rs3093664* with wheezing is mediated in part by its effect on HDL-C levels.

### Evidence for underlying mechanisms of action

*TNF-a* gene codes for a highly pro-inflammatory cytokine [33] involved in many pathophysiological mechanisms of asthma [34, 35]. *TNF-a* is also known to affect lipid metabolism and lipid serum levels [36, 37]. Specific polymorphisms of the *TNFa* gene have been reported to affect HDL-C levels [38] and have been proposed as risk factors towards obesity, metabolic syndrome and type 2 diabetes mellitus [39]. Our findings point towards a novel protective effect of genotype GG of *TNFa rs3093664* against wheezing but also at the same time suggest a strong association of this genotype with higher levels of HDL-C. To date, only few studies have examined the association of this particular polymorphism with asthma [26] or any metabolic condition [40]. Although *TNF-a* serum levels were not assessed in this study, previous reports have documented that *TNF-a* serum levels are inversely associated with serum HDL-C in the general population [41], whereas administration of anti *TNF-a* agents in patients with rheumatic diseases has been shown to result in elevated HDL-C levels [42, 43]. Coupled with these observations, our results suggest that in this small group of carriers of the GG genotype we may have a compromised TNF-a pro-inflammatory activity, manifesting as significantly lower risk for wheezing. At the same time, the direct protective effect of genotype GG for wheezing is further enhanced by the higher HDL-C levels that result from the reduced activity of TNF-a and its inflammatory properties [1].

Enzyme PKC-a has been proposed to be involved in asthma pathogenesis [44], in adipogenesis [45] and lipid mediated insulin resistance [46]. The potential pleiotropic effect of *PRKCA* gene on asthma and BMI was demonstrated by different SNPs influence on asthma or obesity [13, 21], including rs9892651. Our findings also support a pleiotropic effect of *PRKCA* rs9892651 as the minor C allele appeared to be more prevalent among current wheezers and also associated with lower HDL-C levels, although the effect on the risk of wheezing appeared to be very modest.

Polymorphisms of *ADRB2* gene have been studied extensively in asthma but also in lipolysis of the adipose tissue [47]. We examined three of the most well studied polymorphisms of *ADRB2 gene*, the common *rs1042714* and *rs1042713* and the rarer rs1800888 [48, 49]. Our study provides evidence in support of a modest protective role for wheezing of T allele of the rare polymorphism *rs1800888* but also for a modest association with higher HDL-C levels. This is the first study which evaluated the effects of *rs1800888* on HDL-C levels since previous studies mainly examined cardiovascular outcomes with conflicting results [50–52].

### Study limitations

The use of self-reported questionnaire data obtained from a young population to define wheezing and select cases and controls is not as accurate as the use of clinical parameters. We do have clinical phenotypic features of asthma severity in the selected cases populations, as depicted in previous studies [53], but restriction of the analyses to those with features of asthma severity would have reduced the numbers substantially and weaken any possible statistical associations. We think however that any misclassification or recall bias in defining the disease status is unlikely to have been influenced by the genotypes of the examined polymorphisms and thus confound the recovered associations. Furthermore, as five SNPs deviated from HW equilibrium, a random subset (10 %) of samples was genotyped in duplicate for quality control and reproducibility of the genotype calls was confirmed. Populations may deviate from Hardy-Weinberg equilibrium due to selection pressure, structured populations, in-migration, fluctuations in small populations, and if mutations are recent. It might be expected that selection pressure might account for at least some disequilibrium in the disease associated SNPs identified in this study, but other SNPs that did not associate with this particular disease also showed disequilibrium. Even if other disease processes might account for those, this is also a small island population that has experienced multiple historic and recent in-migrations, and which is also a structured population. The number of SNPs studied was limited and although we cannot exclude that other genetic loci that were not tested might also be implicated in the association between wheezing and HDL, this does not affect the validity of the recovered associations. On the other hand, we did investigate several genes/genotypes and although we had some a priori evidence to support our hypotheses, the associations we found do not hold after adjusting for multiple testing by the Bonferroni method, which underlines the need of confirmation of our results in other populations. Finally, this study remains a genetically-focused report without functional phenotypic data on the analyzed SNP variants and HDL-related metabolites. Nevertheless, our novel findings call for further studies to explore the physiological mechanisms behind these associations.

## Conclusions

In conclusion, this study provides a first indication that two SNPs located in different genetic loci, *TNFa rs3093664* and *PRKCA rs9892651,* are associated with both the wheezing and HDL-C levels. The association of *TNFa rs3093664* with wheezing was mediated in part by its effect on HDL-C levels whereas the associations of *PRKCA rs9892651* with wheezing appeared to be independent of HDL-C levels. Further studies are needed to confirm these findings and reveal the functional mechanisms implicated in the interactions of HDL with wheezing and genetic loci expression.

## Abbreviations

ACAS, active asthmatics; ADRB2, β2‐adrenergic receptor; CUWH, current wheezers; HDL-C, high density lipoprotein cholesterol; NWNA, never wheezers never asthmatics; OR, odds ratio; PRKCA, protein kinase C alpha; SNPs, single nucleotide polymorphisms; TNF-a, tumor necrosis factor alpha

## Additional files

Additional file 1: Table S1. Hardy – Weinberg equilibrium and MAF results. (DOCX 15 kb) Additional file 2: Table S2. Genotype distribution of all 16 SNPs in Current Wheezers (CUWH) Vs Controls (NWNA) and HDL-C levels. (DOCX 28 kb) Additional file 3: Table S3. SNPs genotype distribution in Active Asthmatics (ACAS) Vs Controls (NWNA) and HDL-C levels. (DOCX 16 kb) Additional file 4: Table S4. Allelic comparisons for 16 SNPs in Never Wheezers Never Asthmatics (NWNA) Vs Current Wheezers (CUWH) and in Never Wheezers Never Asthmatics (NWNA) Vs Active Asthmatics (ACAS). (DOCX 21 kb)
