# Supplementary material for: Shared genetic variants between serum levels of high-density lipoprotein cholesterol and wheezing in a cohort of children from Cyprus
Source: Ital J Pediatr. 2016 Jul 13;42:67. doi: 10.1186/s13052-016-0276-1 (PMC4944514; doi:10.1186/s13052-016-0276-1)
Supplement: Additional file 1: Table S1. — Hardy – Weinberg equilibrium and MAF results. (DOCX 15 kb) [file 13052_2016_276_MOESM1_ESM.docx]

Supplementary Table 1: Hardy – Weinberg equilibrium and MAF results

| **Gene** | **Chromosome** | **SNPs Genotyped** | **dbSNPrs#** | **Alleles** | **MAF*** | **HW (p_value_)†** |
| --- | --- | --- | --- | --- | --- | --- |
| IL1R1 | 2 | 1 | rs1420101 | C>T | 0.40 | 0.558 |
|  |  |  |  |  |  |  |
| ACP1 | 2 | 1 | rs12714402 | G>A | 0.35 | 0.079 |
|  |  |  |  |  |  |  |
| GNPDA2 | 4 | 1 | rs10938397 | A>G | 0.37 | 0.043 |
|  |  |  |  |  |  |  |
| IL13 | 5 | 1 | rs20541 | C>T | 0.18 | 0.191 |
|  |  |  |  |  |  |  |
| ADRB2 | 5 | 3 | rs1800888  rs1042714  rs1042713 | C>T  C>G  G>A | 0.03  0.30  0.39 | 0.517  0.061  0.052 |
|  |  |  |  |  |  |  |
| TNF-a | 6 | 3 | rs3093664  rs1800629  rs361525 | A>G  G>A  G>A | 0.12  0.09  0.03 | <0.001  0.200  0.390 |
|  |  |  |  |  |  |  |
| LEP | 7 | 1 | rs2167270 | G>A | 0.35 | 0.005 |
|  |  |  |  |  |  |  |
| ACE | 17 | 2 | rs4343  rs4311 | A>G  C>T | 0.33  0.32 | 0.104  <0.001 |
|  |  |  |  |  |  |  |
| GSDMB | 17 | 1 | rs7216389 | T>C | 0.48 | 0.237 |
|  |  |  |  |  |  |  |
| PRKCA | 17 | 2 | rs9892651  rs9901804 | T>C  G>A | 0.39  0.10 | 0.525  0.047 |
|  |  |  |  |  |  |  |
| * MAF: Minor Allele Frequency (control group)  †HW: Chi square test for Hardy-Weinberg (control group) | | | | | | |
