# Supplementary material for: Shared genetic variants between serum levels of high-density lipoprotein cholesterol and wheezing in a cohort of children from Cyprus
Source: Ital J Pediatr. 2016 Jul 13;42:67. doi: 10.1186/s13052-016-0276-1 (PMC4944514; doi:10.1186/s13052-016-0276-1)
Supplement: Additional file 2: Table S2. — Genotype distribution of all 16 SNPs in Current Wheezers (CUWH) Vs Controls (NWNA) and HDL-C levels. (DOCX 28 kb) [file 13052_2016_276_MOESM2_ESM.docx]

Supplementary table 2: Genotype distribution of all 16 SNPs in Current Wheezers (CUWH) Vs Controls (NWNA) and HDL-C levels.

| **Gene** | **SNP (genotype)** | **NWNA (n) (%)** | **CUWH (n) (%)** | **χ^2^*** | ***P* value** | ***P***  **trend** | **HDL mg/dl**  **(mean & 95% CI)** | ***F*†** | ***P* value** | ***P***  **trend** |
| --- | --- | --- | --- | --- | --- | --- | --- | --- | --- | --- |
|  |  | | | | | | | | | |
| ***IL1R1*** | ***rs1420101*** |  |  |  |  |  |  |  |  |  |
|  | **CC** | 186 (35%) | 55 (34.6%) |  |  |  | 52.62(50.81-54.44) |  |  |  |
|  | **CT** | 262 (49.3%) | 77 (48.4%) |  |  |  | 54.23(52.74-55.73) |  |  |  |
|  | **TT** | 83 (15.6%) | 27 (17%) | 0.167 | 0.920 | 0.774 | 53.47(51.08-56.47) | 0.919 | 0.339 | 0.321 |
|  |  |  |  |  |  |  |  |  |  |  |
| **ACP1** | ***rs12714402*** |  |  |  |  |  |  |  |  |  |
|  | **AA** | 68 (10.7%) | 25 (13.8%) |  |  |  | 54.03 (51.17-56.89) |  |  |  |
|  | **AG** | 310 (48.7%) | 79 (43.6%) |  |  |  | 53.91 (52.52-55.30) |  |  |  |
|  | **GG** | 259 (40.7%) | 77 (42.5%) | 2.088 | 0.352 | 0.822 | 52.74(51.29-54.18) | 0.751 | 0.472 | 0.262 |
|  |  |  |  |  |  |  |  |  |  |  |
| ***GNDPA2*** | ***rs10938397*** |  |  |  |  |  |  |  |  |  |
|  | **AA** | 263 (41.2%) | 60 (33.9%) |  |  |  | 53.32 (51.82-54.82) |  |  |  |
|  | **AG** | 275 (43%) | 86 (48.6%) |  |  |  | 54.22 (52.79-55.66) |  |  |  |
|  | **GG** | 101 (15.8%) | 31 (17.5%) | 3.061 | 0.216 | 0.137 | 52.21 (49.89-54.53) | 1.106 | 0.331 | 0.704 |
|  |  |  |  |  |  |  |  |  |  |  |
| ***IL13*** | ***rs20541*** |  |  |  |  |  |  |  |  |  |
|  | **CC** | 419 (66.2%) | 111 (64.9%) |  |  |  | 53.79 (52.60-54.95) |  |  |  |
|  | **CT** | 198 (31.3%) | 53 (31.0%) |  |  |  | 53.28(51.61-54.95) |  |  |  |
|  | **TT** | 16 (2.5%) | 7 (4.1%) | 1.191 | 0.551 | 0.540 | 49.55 (43.76-55.34) | 1.107 | 0.331 | 0.253 |
|  |  |  |  |  |  |  |  |  |  |  |
| ***ADRB2*** | ***rs1800888*** |  |  |  |  |  |  |  |  |  |
|  | **CC** | 609 (95%) | 175 (97.8%) |  |  |  | 53.3 (52.3-54.2) |  |  |  |
|  | **CT** | 32 (5.0%) | 4 (2.2%) | 2.535 | 0.076^‡^ | - | 57.2 (51.7-62.8) | 2.840 | 0.092 | - |
|  |  |  |  |  |  |  |  |  |  |  |
| **ADRB2** | ***rs1042714*** |  |  |  |  |  |  |  |  |  |
|  | **CC** | 312 (50.4%) | 89 (51.1%) |  |  |  | 53.47 (52.21-54.74) |  |  |  |
|  | **CG** | 241 (38.9%) | 70 (40.2% |  |  |  | 52.77 (51.16-54.38) |  |  |  |
|  | **GG** | 66 (10.7%) | 15 (8.6%) | 0.627 | 0.731 | 0.627 | 55.11 (51.59-58.64) | 0.949 | 0.388 | 0.701 |
|  |  |  |  |  |  |  |  |  |  |  |
| **ADRB2** | **rs1042713** |  |  |  |  |  |  |  |  |  |
|  | **AA** | 103 (16.9%) | 26 (15.7%) |  |  |  | 53.18 (50.80-55.57) |  |  |  |
|  | **AG** | 266 (43.8%) | 74 (44.6%) |  |  |  | 53.15 (51.67-54.63) |  |  |  |
|  | **GG** | 239 (39.3%) | 66 (39.8%) | 0.155 | 0.925 | 0.782 | 54.12 (52.25-55.29) | 0.446 | 0.640 | 0.416 |
|  |  |  |  |  |  |  |  |  |  |  |
| **TNFa** | ***rs3093664*** |  |  |  |  |  |  |  |  |  |
|  | **AA** | 500 (79.6%) | 134 (76.1%) |  |  |  | 53.3 (52.2-54.3) |  |  |  |
|  | **AG** | 102 (16.2%) | 40 (22.7%) |  |  |  | 51.7 (49.4-54.1) |  |  |  |
|  | **GG** | 26 (4.1%) | 2 (1.1%) | 7.047 | 0.03 | 0.912 | 65.3 (59.2-71.4) | 11.787 | >0.001 | 0.028 |
|  |  |  |  |  |  |  |  |  |  |  |
| ***TNFa*** | ***rs1800629*** |  |  |  |  |  |  |  |  |  |
|  | **AA** | 2 (0.3%) | 0 (0%) |  |  |  | 82.00 (82.00-82.00) |  |  |  |
|  | **AG** | 102 (16.3%) | 21 (12.2%) |  |  |  | 53.46 (51.13-55.79) |  |  |  |
|  | **GG** | 522 (83.4%) | 151 (87.8%) | 2.320 | 0.313 | 0.141 | 53.24 (52.30-54.39) | 3.734 | 0.024 | 0.476 |
|  |  |  |  |  |  |  |  |  |  |  |
| **TNFa** | rs361525 |  |  |  |  |  |  |  |  |  |
|  | **AA** | 0 (0%) | 1 (0.6%) |  |  |  | 56.00() |  |  |  |
|  | **AG** | 41 (6.7% | 10 (5.8%) |  |  |  | 54.52 (50.59-58.46) |  |  |  |
|  | **GG** | 567 (93.3%) | 161 (93.6%) | 3.714 | 0.156 | 0.916 | 53.20 (52.19-54.21) | 0.236 | 0.790 | 0.493 |
|  |  |  |  |  |  |  |  |  |  |  |
| **LEP** | **rs2167270** |  |  |  |  |  |  |  |  |  |
|  | **AA** | 95 (14.9%) | 25 (14.1%) |  |  |  | 54.65 (51.94-57.36) |  |  |  |
|  | **AG** | 258 (40.5%) | 76 (42.9%) |  |  |  | 54.06 (52.52-55.60) |  |  |  |
|  | **GG** | 284 (44.6%) | 76 (42.9%) | 0.344 | 0.842 | 0.887 | 52.40 (51.09-53.71) | 1.843 | 0.159 | 0.065 |
|  |  |  |  |  |  |  |  |  |  |  |
| **ACE** | rs4343 |  |  |  |  |  |  |  |  |  |
|  | **GG** | 74 (11.7%) | 24 (13.3%) |  |  |  | 53.93 (50.96-56.90) |  |  |  |
|  | **GA** | 252 (42.9%) | 75 (41.7%) |  |  |  | 54.16 (52.27-55.65) |  |  |  |
|  | **AA** | 288 (45.4%) | 81 (45%) | 0.377 | 0.828 | 0.717 | 52.74 (51.40-54.08) | 1.007 | 0.366 | 0.232 |
|  |  |  |  |  |  |  |  |  |  |  |
| **ACE** | rs4311 |  |  |  |  |  |  |  |  |  |
|  | **TT** | 4 (0.6%) | 2 (1.1%) |  |  |  | 54.28 (39.43-69.13) |  |  |  |
|  | **TC** | 407 (63.4%) | 114 (63%) |  |  |  | 53.52 (52.35-54.70) |  |  |  |
|  | **CC** | 231 (36) | 65 (35.9%) | 0.454 | 0.797 | 0.894 | 53.47 (51.88-55.06) | 0.011 | 0.989 | 0.933 |
|  |  |  |  |  |  |  |  |  |  |  |
| **GSDMB** | rs7216389 |  |  |  |  |  |  |  |  |  |
|  | **CC** | 154 (24.1%) | 39 (21.8%) |  |  |  | 54.20 (52.20-56.20) |  |  |  |
|  | **CT** | 304 (47.6%) | 89 (49.7%) |  |  |  | 53.51 (52.15-54.88) |  |  |  |
|  | **TT** | 181 (28.3%) | 51 (28.5%) | 0.452 | 0.798 | 0.684 | 52.95 (51.22-54.68) | 0.437 | 0.646 | 0.352 |
|  |  |  |  |  |  |  |  |  |  |  |
| ***PRKCA*** | ***rs9892651*** |  |  |  |  |  |  |  |  |  |
|  | **TT** | 230 (37.0) | 52 (30.2) |  |  |  | 55.2 (53.4-56.9) |  |  |  |
|  | **CT** | 302 (48,6) | 87 (50.6) |  |  |  | 52.7 (51.4-54.1) |  |  |  |
|  | **CC** | 89 (14.3) | 33 (19.2) | 3.921 | 0.141 | 0.048 | 52.2 (50.0-54.5) | 3.179 | 0.042 | 0.020 |
|  |  |  |  |  |  |  |  |  |  |  |
| ***PRKCA*** | rs9901804 |  |  |  |  |  |  |  |  |  |
|  | **AA** | 2 (0.3%) | 2 (1.1%) |  |  |  | 50.37 (35.68-65.06) |  |  |  |
|  | **AG** | 125 (19.7%) | 36 (20.2%) |  |  |  | 53.08 (51.10-55.05) |  |  |  |
|  | **GG** | 507 (80%) | 140 (78.7%) | 1.891 | 0.388 | 0.549 | 53.49 (52.41-54.57) | 0.168 | 0.845 | 0.637 |
|  |  |  |  |  |  |  |  |  |  |  |
|  | * χ^2^ test (asymptomatic 2- sided significance) † One-Way Anova test for equality of means  ‡ Fischer Exact test (exact 1-sided significance) | | | | | | | | | |
