# Supplementary material for: Shared genetic variants between serum levels of high-density lipoprotein cholesterol and wheezing in a cohort of children from Cyprus
Source: Ital J Pediatr. 2016 Jul 13;42:67. doi: 10.1186/s13052-016-0276-1 (PMC4944514; doi:10.1186/s13052-016-0276-1)
Supplement: Additional file 3: Table S3. — SNPs genotype distribution in Active Asthmatics (ACAS) Vs Controls (NWNA) and HDL-C levels. (DOCX 16 kb) [file 13052_2016_276_MOESM3_ESM.docx]

Supplementary Table 3: SNPs genotype distribution in Active Asthmatics (ACAS) Vs Controls (NWNA) and HDL-C levels.

| **SNP (genotype)** | **NWNA**  **(n) (%)** | **ACAS**  **(n) (%)** | **χ^2^** | **p value** | ***p***  **trend** | **HDL mg/dl**  **(mean**  **95% CI)** | ***F*** | **p value** | ***p***  **trend** |
| --- | --- | --- | --- | --- | --- | --- | --- | --- | --- |
| *TNFa* *rs3093664* |  |  |  |  |  |  |  |  |  |
| **AA** | 500(79.6) | 55(85.9) |  |  |  | 53.8  (52.6-54.9) |  |  |  |
| **AG** | 102 (16.2) | 9(14.1) |  |  |  | 52.9  (50.1-55.8) |  |  |  |
| **GG** | 26(4.1) | 0(0) | 3.111 | 0.211^a^ | 0.115 | 66.0  (59.5-72.6) | 10.050 | <0.001 | 0.009 |
|  | | | | | | | | | |
| *PRKCA rs9892651* |  |  |  |  |  |  |  |  |  |
| **TT** | 230 (37.0) | 24 (38.7) |  |  |  | 55.3  (53.4-57.2) |  |  |  |
| **CT** | 302(48.6) | 27 (43.5) |  |  |  | 53.7  (52.2-55.2) |  |  |  |
| **CC** | 89 (8) | 11(17.7) | 0.793 | 0.673^a^ | 0.849 | 53.0  (50.5-55.5) | 1.348 | 0.261 | 0.111 |
|  | | | | | | | | | |
| *ADRB2 rs1800888* |  |  |  |  |  |  |  |  |  |
| **CC** | 609 (95%) | 63 (100%) |  |  |  | 53.9  (52.9-55.0) |  |  |  |
| **CT** | 32 (5%) | 0 (0%) | 3.295 | 0.046^b^ | - | 57.7  (51.7-63.8) | 2.196 | 0.139 | - |
|  |  |  |  |  |  |  |  |  |  |
| ^a^ χ^2^ test (asymptomatic 2- sided significance), ^b^ Fischer Exact test (exact 1-sided significance) | | | | | | | | | |
