# Supplementary material for: Shared genetic variants between serum levels of high-density lipoprotein cholesterol and wheezing in a cohort of children from Cyprus
Source: Ital J Pediatr. 2016 Jul 13;42:67. doi: 10.1186/s13052-016-0276-1 (PMC4944514; doi:10.1186/s13052-016-0276-1)
Supplement: Additional file 4: Table S4. — Allelic comparisons for 16 SNPs in Never Wheezers Never Asthmatics (NWNA) Vs Current Wheezers (CUWH) and in Never Wheezers Never Asthmatics (NWNA) Vs Active Asthmatics (ACAS). (DOCX 21 kb) [file 13052_2016_276_MOESM4_ESM.docx]

Supplementary Table 4: Allelic comparisons for 16 SNPs in Never Wheezers Never Asthmatics (NWNA) Vs Current Wheezers (CUWH) and in Never Wheezers Never Asthmatics (NWNA) Vs Active Asthmatics (ACAS).

| **Gene, SNP (alleles)** | **NWNA**  **(n) (%)** | **CUWH**  **(n) (%)** | **OR (95% CI)** | ***P* value** | ***ACAS***  ***(n) (%)*** | ***OR*** | ***P value*** |
| --- | --- | --- | --- | --- | --- | --- | --- |
| ***ACP1 rs12714402*** |  |  |  |  |  |  |  |
| **A** | 446(35%) | 129 (36%) |  |  | 42 (32.8%) |  |  |
| **G** | 828 (65%) | 233 (64%) | 1.028 (0.805-1.312) | 0.823 | 86 (67.2%) | 0.907 (0.616-1.334) | 0.617 |
|  |  |  |  |  |  |  |  |
| ***ADRB2 rs1042714*** |  |  |  |  |  |  |  |
| **C** | 865 (69.9%) | 248 (71.3%) |  |  | 88 (71%) |  |  |
| **G** | 373 (30.1%) | 100 (28.7% | 1.069 (0.823-1.390) | 0.610 | 36 (29%) | 1.054 (0.702 -1.582) | 0.80 |
|  |  |  |  |  |  |  |  |
| ***TNFa rs361525*** |  |  |  |  |  |  |  |
| **A** | 41 (3.4%) | 12 (3.5%) |  |  | 1 (0.8%) |  |  |
| **G** | 1175 (96.6%) | 332 (96.5%) | 1.036 (0.538-1.994) | 0.92 | 121 (99.2%) | 0.237 (0.032-1.737) | 0.156 |
|  |  |  |  |  |  |  |  |
| ***IL1R1 rs1420101*** |  |  |  |  |  |  |  |
| **C** | 634 (59.7%) | 187 (58.8%) |  |  | 65 (56%) |  |  |
| **T** | 428 (40.3%) | 131 (41.2%) | 0.964 (0.747-1.243) | 0.777 | 51 (44%) | 0.86 (0.5845-1.266) | 0.446 |
|  |  |  |  |  |  |  |  |
| ***ACE rs4311*** |  |  |  |  |  |  |  |
| **T** | 415 (32.3%) | 118 (32.6%) |  |  | 44 (34.4%) |  |  |
| **C** | 869 (67.7%) | 244 (67.4%) | 1.013 (0.789-1.299) | 0.920 | 84 (65.6%) | 1.097 (0.748-1.609) | 0.639 |
|  |  |  |  |  |  |  |  |
| ***ACE rs4343*** |  |  |  |  |  |  |  |
| **G** | 400 (32.6%) | 123 (34.2%) |  |  | 50 (39%) |  |  |
| **A** | 828 (67.4%) | 237 (65.8%) | 1.074 (0.838-1.377) | 0.572 | 78 (61%) | 1.327 (0.912-1.930) | 0.138 |
|  |  |  |  |  |  |  |  |
| ***GNDPA2 rs10938397*** |  |  |  |  |  |  |  |
| **A** | 801 (62.7%) | 206 (58.2%) |  |  | 70 (54.7%) |  |  |
| **G** | 477 (37.3%) | 148 (41.8%) | 0.829 (0.652-1.053) | 0.124 | 58 (45.3%) | 0.719 (0.498-1.036) | 0.076 |
|  |  |  |  |  |  |  |  |
| ***TNFa rs1800629*** |  |  |  |  |  |  |  |
| **A** | 106 (8.5%) | 21 (6.1%) |  |  | 9 (7.3%) |  |  |
| **G** | 1146 (91.5%) | 323 (93.9%) | 0.703 (0.433-1.141) | 0.151 | 115 (92.7%) | 0.846 (0.417-1.716) | 0.639 |
|  |  |  |  |  |  |  |  |
| ***GSDMB rs7216389*** |  |  |  |  |  |  |  |
| **C** | 612 (47.9%) | 167 (46.6%) |  |  | 54 (42.9%) |  |  |
| **T** | 666 (52.1%) | 191 (53.4%) | 0.951 (0.752-1.203) | 0.680 | 72 (57.1%) | 0.816 (0.564-1.181) | 0.281 |
|  |  |  |  |  |  |  |  |
| ***LEP rs2167270*** |  |  |  |  |  |  |  |
| **A** | 448 (35.2%) | 126 (35.6%) |  |  | 44 (28.9%) |  |  |
| **G** | 826 (64.8%) | 228 (64.4%) | 1.019 (0.797-1.303) | 0.888 | 108 (71.1%) | 0.751 (0.519-1.086) | 0.128 |
|  |  |  |  |  |  |  |  |
| ***PRKCA rs9901804*** |  |  |  |  |  |  |  |
| **A** | 129 (10.2%) | 40 (11.2%) |  |  | 10 (8.1%) |  |  |
| **G** | 1139 (89.8%) | 316 (88.8%) | 1.118 (0.767-1.628) | 0.560 | 114 (91.9%) | 0.774 (0.396-1.516) | 0.454 |
|  |  |  |  |  |  |  |  |
| ***ADRB2 rs1042713*** |  |  |  |  |  |  |  |
| **A** | 472 (38.8%) | 126 (38%) |  |  | 44 (37.3%) |  |  |
| **G** | 744 (61.2%) | 206 (62%) | 0.964 (0.751-1.238) | 0.777 | 74 (62.7%) | 0.937 (0.634-1.385) | 0.740 |
|  |  |  |  |  |  |  |  |
| ***IL13 rs20541*** |  |  |  |  |  |  |  |
| **C** | 1036 (81.8%) | 275 (80.4%) |  |  | 98 (77.8%) |  |  |
| **T** | 230 (18.2%) | 67 (19.6%) | 0.911 (0.673-1.233) | 0.549 | 28 (62.7%) | 0.777 (0.499-1.211) | 0.264 |
|  |  |  |  |  |  |  |  |
| ***TNFa rs3093664*** |  |  |  |  |  |  |  |
| **A** | 1102 (87.7%) | 308 (87.5%) |  |  | 119 (93%) |  |  |
| **G** | 154(12.3%) | 44 (12.5%) | 0.978 (0.684-1.399) | 0.920 | 9 (7%) | 1.848 (0.919-3.714) | 0.080 |
|  |  |  |  |  |  |  |  |
| ***PRKCA rs9892651*** |  |  |  |  |  |  |  |
| **T** | 762 (61.4%) | 191 (55.5%) |  |  | 75 (60.5%) |  |  |
| **C** | 480 (38.6%) | 153 (44.5%) | 0.786 (0.618-1.001) | 0.051 | 49 (39.5%) | 0.964 (0.661-1.407) | 0.841 |
|  |  |  |  |  |  |  |  |
| ***ADRB2 rs1800888*** |  |  |  |  |  |  |  |
| **C** | 1250 (97.5%) | 359 (98.9%) |  |  | 126 (100%) |  |  |
| **T** | 32 (2.5%) | 4 (1.1%) | 2.266 (0.796–6.449) | 0.115 | 0 (0%) | - | - |
